# Exploring freshwater stream bacterial communities as indicators of land use intensity

**DOI:** 10.1186/s40793-024-00588-z

**Published:** 2024-07-08

**Authors:** Syrie Hermans, Anju Gautam, Gillian D. Lewis, Martin Neale, Hannah L. Buckley, Bradley S. Case, Gavin Lear

**Affiliations:** 1 School of Science, Auckland University of Technology, 34 St Paul Street, Auckland, 1142 New Zealand; 2 School of Biological Sciences, The University of Auckland, 3a Symonds Street, Auckland, 1010 New Zealand; 3 Puhoi Stour, 15 Taipari Road, Te Atatu, Auckland, 0610 New Zealand

## Abstract

**Background:**

Stream ecosystems comprise complex interactions among biological communities and their physicochemical surroundings, contributing to their overall ecological health. Despite this, many monitoring programs ignore changes in the bacterial communities that are the base of food webs in streams, often focusing on stream physicochemical assessments or macroinvertebrate community diversity instead. We used 16S rRNA gene sequencing to assess bacterial community compositions within 600 New Zealand stream biofilm samples from 204 sites within a 6-week period (February–March 2010). Sites were either dominated by indigenous forests, exotic plantation forests, horticulture, or pastoral grasslands in the upstream catchment. We sought to predict each site’s catchment land use and environmental conditions based on the composition of the stream bacterial communities.

**Results:**

Random forest modelling allowed us to use bacterial community composition to predict upstream catchment land use with 65% accuracy; urban sites were correctly assigned 90% of the time. Despite the variation inherent when sampling across a ~ 1000-km distance, bacterial community data could correctly differentiate undisturbed sites, grouped by their dominant environmental properties, with 75% accuracy. The positive correlations between actual values and those predicted by the models built using the stream biofilm bacterial data ranged from weak (average log N concentration in the stream water, R^2^ = 0.02) to strong (annual mean air temperature, R^2^ = 0.69).

**Conclusions:**

Freshwater bacterial community data provide useful insights into land use impacts on stream ecosystems; they may be used as an additional measure to screen stream catchment attributes.

**Supplementary Information:**

The online version contains supplementary material available at 10.1186/s40793-024-00588-z.

## Introduction

Land use transitions from natural to managed land often degrade ecosystem health [53, 58]. Anthropogenic land uses can impact catchment soil characteristics and hydrological regimes, as well as conditions within receiving waterways such as pH, temperature, light exposure and the availability of carbon and nutrients, all of which impact stream communities [13, 16, 28, 29, 55]. Reflecting this, stream and catchment ecological health is frequently monitored and even quantified by assessing the abundances and compositions of taxa, including fish and macroinvertebrates within waterways [17]. Other organisms, such as diatoms [10] and algae, [51] have also been highlighted as valuable indicators of stream quality. However, these traditional methods for monitoring, for example, using invertebrates, involve the time-consuming and challenging tasks of collecting and identifying individual specimens [57]. In the face of ever-changing environmental conditions, and growing populations leading to increased anthropogenic impacts on increasing numbers of waterways, more robust, high throughput and time-efficient monitoring tools are needed.

To try meet these demands, more recently, substantial new opportunities have arisen for using microbial DNA data to explore catchment land management impacts on aquatic microbial communities [18]. While bacterial DNA data is not routinely used to monitor freshwater, there is evidence that bacterial communities reflect land use [9] and catchment disturbance to similar degrees as both traditional macroinvertebrate community indicator data and abiotic water quality data [27]. Further, the potential of microbial communities to act in this capacity has been demonstrated in other environments. Machine learning approaches, such as random forest analysis, have been used by researchers, including Good et al. [20] and Glasl et al. [19], to determine the potential of bacterial communities as bioindicators of environmental perturbations, predicting seasonal discharge dynamics and assessing environmental change over space and time. Likewise, Hermans et al. [23] showed that soil microbial community DNA can be used to predict the dominant land use of a samples origin (e.g., exotic forest, indigenous forest, horticulture, or dairy), specific soil environmental variables, and overall soil health status.

To assess the potential of bacterial community data to contribute to stream monitoring, similar to how macroinvertebrate data has been used for decades [15], we analysed bacterial 16S rRNA gene sequence data from 204 stream sites draining rural, urban, exotic and native forest-dominated catchments throughout New Zealand, sampled during February–March 2010. A land use index score was generated based on the different land use intensities in the upstream catchment, and extensive metadata was collected or collated. With these data, we used machine-learning models to associate bacterial community relationships with a broad suite of catchment-related environmental conditions.

## Methods

### Experimental outline

This study used the same sample and DNA sequence data reported by Lear et al. [29], comprising a subset of the 244 sites originally sampled by Lear et al. [28]. Stream biofilm samples were analysed from 204 sites across multiple regions of Aotearoa-New Zealand, ranging from Canterbury (South Island) to Auckland (North Island) during the Austral summer (February–March 2010, Fig. S1). Catchment land use data, provided by each local authority at the time of sampling, classifies each site as rural (n = 106), urban (n = 25), exotic forest (n = 11) or native forest (n = 62) based on the dominant land cover upstream [40]. Further, attempting to quantify the impacts of anthropogenic land uses across each catchment, we calculated a land use index (LUI) value for each site using the equation ‘4 × urban + 2 × crop + 1 × pasture’ and adding + 1 to each site to eliminate the presence of any zero values, for equal and comparable interpretation. Upstream catchment areas were visually delineated and digitized as polygons within ArcGIS 9.3 [12] using a river layer from the New Zealand REC system [50]. The terms ‘urban’, ‘crop’ and ‘pasture’ refer land use percentage in the catchment upstream, with exotic forests treated as a ‘crop’ for the purposes of this index. Higher LUI values are then expected to represent more degraded catchments and were used to categorise streams into four different LUI ranges: very high (1; 400–200), high (2; 199–100), low (3; 99–50) and very low (4; 49–1; Table S1).

Soil physicochemical parameters and land use characteristics of the upstream catchments were previously collated for each site using ArcGIS, as described in Lear et al. [28]. Briefly, site elevation data were extracted from a 25 m resolution digital elevation model (DEM) [3]. Mean annual solar radiation, air temperature, precipitation and seasonal precipitation variation (average difference between total precipitation in summer and winter) of each stream samples location were extracted from NIWA’s long-term climate database [59]. Once upstream catchment polygons were digitised as polygons [28], data for each catchment were extracted using GIS overlay methods, including (1) land cover (LCDB2; MfE [35]), (2) annual average concentration of total nitrogen in the water (log ppb) using the FENZ database [30], (c) soil pH and total carbon (%, 0.2 m depth) using the NZLRI database [41]. These data (excluding the land cover data) were reduced via principal components analysis, using the rda() function to analyse normalised data in the ‘vegan’ package within R [45]; for subsequent analyses, the first two axes were extracted.

### Sample collection

We scraped biofilm from the surface of five rocks at each site by abrasion with sterile sponges (Speci-Sponge; VWR International Ltd., IL., USA) as described in Lear et al. [28]. Sponges were placed into separate bags (Whirl–Pak, VWR International), sealed and chilled (− 20 °C).

### Sample processing

Sponges were immersed in sterile water and macerated using a Stomacher 400 device (Seward, Norfolk, UK) at high speed for 90 s, to separate the biofilm samples. Sponges were squeezed to remove biofilm material, which was then pelleted by centrifugation (8000×*g*, 20 min). We used the approach of Miller et al. [36] to remove DNA from each pelleted sample. To characterise the bacterial community composition at each site, the hypervariable region V4 of bacterial 16S rRNA genes was amplified using 515f/806r primers [7], which we altered to include Illumina flow cell adaptor sequences. Reverse primers, specific to each sample, contained DNA barcodes unique to each sample for sample multiplexing [7]. PCR was done in triplicate as detailed by Lear et al. [29] before products were pooled and purified with SequalPrep Normalization kits (Invitrogen, New Zealand). Extracts were run on an Illumina MiSeq sequencing machine using 2 × 150 bp chemistry.

### Bioinformatics and statistical analyses

All bioinformatic and statistical analyses were performed in R v 4.2.2 [45]. DADA2 v 1.26.0 (Callahan et al., 2016) was used for quality filtering, denoising and amplicon sequence variant (ASV) inference, chimaera removal and taxonomic assignment. Primer sequences were removed from reads, and then reads were truncated (to 140 bp). Reads with greater than two (forward reads) or three (reverse reads) expected errors were removed, and reads truncated at the first instance where the quality score was ≤ 2. After applying the DADA2 core algorithm to call ASVs, forward and reverse reads were merged. Chimeric ASVs were removed before taxonomic assignment against the Silva v 138.1 taxonomic reference database [44]. Replicate samples (n = 3) were merged for each site, resulting in 204 samples, each representing the bacterial community at one site. ASVs that were not classified as bacterial or were classified as being of mitochondrial or chloroplast origin were removed. CSS normalisation could not improve the fold-difference in sequencing depth, so rarefaction was used instead. For this, the ‘rarefy_even_depth’ function from the phyloseq package v 1.42.0 [34] was used, with a sample size of 23,718. Three subsets of the data were created: one containing all samples (n = 204), one containing all disturbed sample data (exotic forests, urban and rural; n = 142) and one containing all data from undisturbed sites (native forests; n = 62) (Table S2).

The ASV tables were processed to reduce numbers of explanatory variables used for the random forest models. ASVs with a total abundance of less than ten across all samples were removed before sites were clustered based on ASV abundances using the ‘NbClust’ command from the package of the same name (v 3.0.1; [8]). A Bray–Curtis matrix created using the ‘vegan’ package v 2.6.4 [42] was used as the input, Ward’s minimum variance clustering was used, and silhouettes were used as the index to select the best number of clusters. Subsequently, indicator taxa, representing the communities of each subcluster, were determined using the ‘multipatt’ command from the ‘indicspecies’ package v 1.7.14 [6] with default parameters. We then selected indicator ASVs based on them having a minimum positive predictive value (At) and a minimum sensitivity value (Bt) of 0.5. All ASVs highlighted as indicators for one or more clusters using these parameters were then selected for subsequent analysis, meaning their abundance across all sites, not just the ones for which they were indicators, was used. This resulted in three ASV tables: (1) an ASV table containing 226 ASVs best representing the variation across all 204 sites, (2) an ASV table containing 183 ASVs best representing the variation across the 142 disturbed sites, and (3) an ASV table containing 397 ASVs best representing the variation across the 62 undisturbed sites (Table S2).

Bacterial community composition differences between catchments were visualised with nMDS plots for both the subset of ASVs obtained as described above, and the full dataset, using Vegan’s ‘metaMDS’ command with Bray–Curtis as the clustering method and 999 permutations. Vegan’s ‘envfit’ was used to overlay the environmental vectors onto the ordination. Vegan’s ‘mantel’ command was used to check for correlation between the two dissimilarity matrices. Significant differences among data group centroids were analysed by PERMANOVA [2] with 999 permutations using vegan’s ‘adonis’ function in R. The ‘betadispr’ function was used to analyse multivariate homogeneity in data group dispersions and ‘permutest’ was used to determine if the homogeneity of the multivariate dispersion was significantly different between catchments.

We used random forest analyses to assess the ability of the ASV subsets to classify, or predict, different qualitative and quantitative characteristics of the sites, as detailed below. The ‘randomForest’ package v 4.7.1.1 [32] and command of the same name were used for each random forest model, with default parameters. Before running the models, the sites were divided into ‘training’ and ‘validation’ subsets. For this, stratified sampling was used to randomly assign 80% of the sites as the training dataset, and the remaining 20% were selected for validating the model. Variables used for the stratified sampling varied for the different models and are outlined in Table S2. To avoid spurious results, the stratified subsampling and modelling were repeated for 100 random iterations for each model, and the results were combined into one. All the visualised results are for the validation subset.

Classification models were used to predict categorical response variables, based on the composition of the representative ASVs. For the dataset containing all sites, the variable modelled was the catchment type assigned to each site. Additional variables modelled were each site's land use index score, the ‘land use cluster’ of each site, and the ‘environmental cluster’ of each site. Finally, for the undisturbed sites, we modelled the ability of the ASV data to predict the ‘environmental cluster’ of each site. For the land use and environmental clusters, sites were assigned to these clusters using the same Ward’s minimum variance clustering as described above, except this time, clustering was based on the Euclidean distances of either the land use index and catchment land use composition (% of cover) for the land use clusters, or catchment-scale environmental data of the upstream catchments for the environmental clusters (Table S2). Dunn’s tests, performed using the ‘dunn.test’ package v 1.3.5 [11], were used to determine how the variables used during the clustering differed between the different clusters, and Principal Component Analysis (PCA) conducted with Vegan’s ‘rda’ function was used to visualise the underlying differences among sites. The same variables were used in the clustering to generate the PCA scores.

Regression models were used to predict continuous response variables based on the composition of the representative ASVs for the disturbed and undisturbed datasets (Table S2). For this, we assessed the ability of ASVs to correctly predict the mean precipitation, variation in precipitation, average stream N, mean soil C, median soil pH, mean annual solar radiation (MASR), mean annual temperature (MAT) and elevation. R^2^ values and the regression slope between the predicted and actual values were used to determine the accuracy of the models.

Finally, the five most important ASVs, determined as the largest mean decrease in accuracy associated with the ASV being excluded (for classification tree analyses) or greatest increase in mean squared error (MSE) associated with the ASV (for regression tree analysis), were identified for each iteration (n = 100) of each model. These were then visualised to determine taxonomic patterns in ASVs deemed most important for improving the accuracy of each model.

## Results

### Stream bacterial community composition across all catchment types

**Fig. 1:**
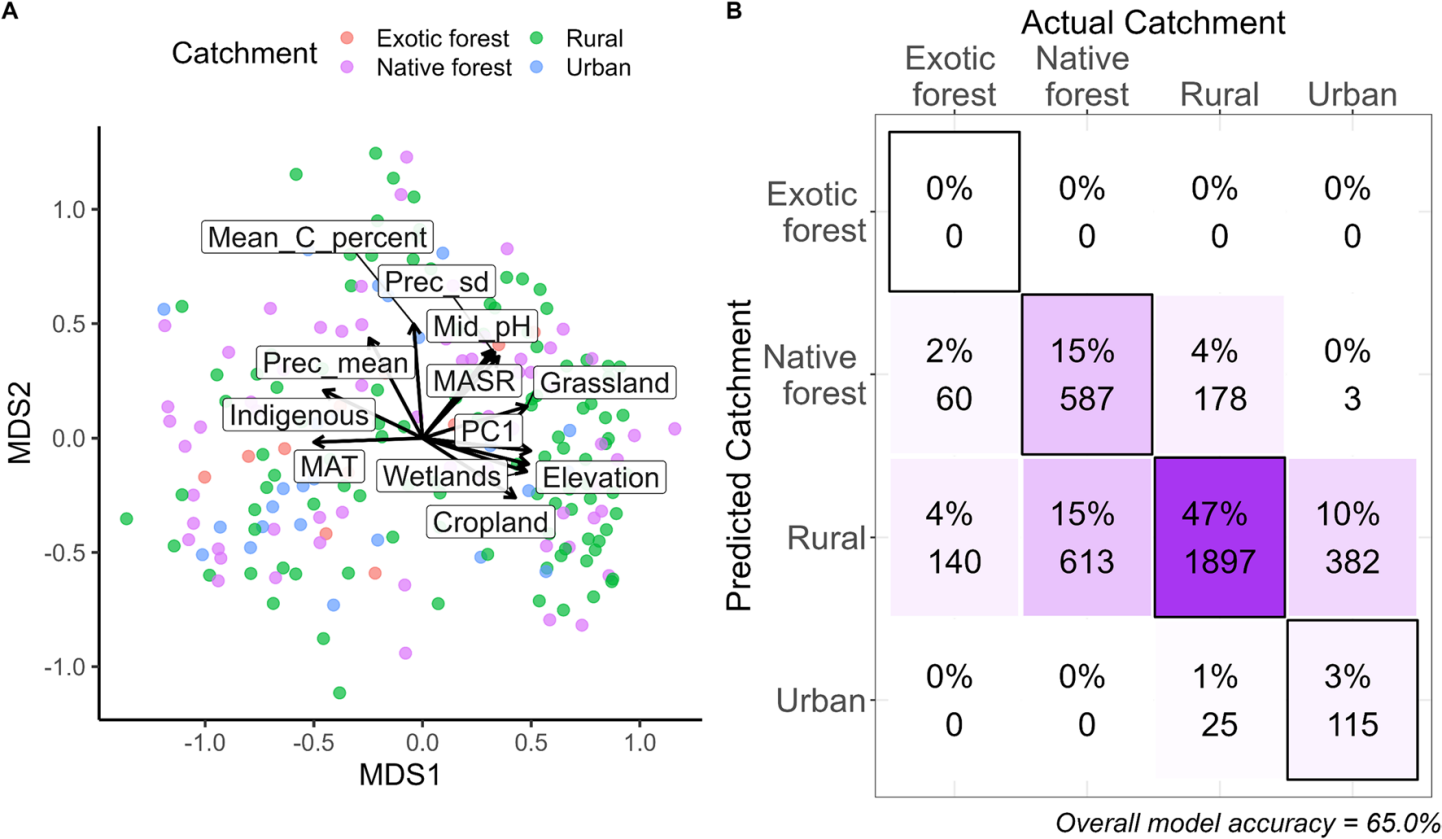
**a** Underlying differences (Bray Curtis dissimilarity) in the subset of ASVs selected as representative of the biological variation across stream biofilm samples collected from urban, rural, exotic- or native-forest catchments. Vectors represent land use and physiochemical variables significantly correlated (*P* < 0.05) with the observed variation in bacterial community composition, as determined by “envfit” analysis of the data; abbreviations are detailed in the supplementary material. **b** The number of correct and incorrect identifications of catchment types (all sites) based on 100 iterations of random forest classification of this bacterial community data. Black borders indicate correct classifications. Both proportions (percentages) and counts are given

After rarefaction, we obtained 47,498 ASVs across the 204 samples. To reduce the number of explanatory variables used for the random forest models, 226 ASVs were selected to represent the biological variation across samples. The strong correlation between Bray–Curtis dissimilarity matrices for the complete dataset (i.e. all ASVs; Fig. S2) to the reduced dataset (Mantel R = 0.92, *P* < 0.001) confirms that the majority of the biological variation was captured in this subsample of ASVs (Fig. S3). When comparing bacterial community data using a Bray–Curtis dissimilarity matrix for this subset of ASVs, bacterial community composition was significantly different among the catchment land uses (PERMANOVA R^2^ = 0.043, *P* < 0.001). However, these differences are at least partly due to differences in dispersion, which also varied significantly for the different catchment land uses (betadisper *P* < 0.01). All combinations of pairwise comparisons showed significant differences in dispersion (Tukey’s P < 0.05) except when comparing exotic forest catchments to urban catchments (*P* = 0.84) and rural catchments to indigenous forest catchments (*P* = 0.95). Indeed, non‐metric multidimensional scaling (nMDS) ordination revealed no strong clustering in the composition of stream biofilm bacterial communities. Still, soil physicochemical variables and upstream percentage catchment land use characteristics were significantly correlated with underlying differences in bacterial community composition (Fig. 1). Variation along the first nMDS axis was driven by climatic variables, soil characteristics such as pH, the proportion of grasslands and wetlands in the upstream catchment, and elevation (Fig. 1a). Variation along the second nMDS axis was primarily correlated with the carbon content, and the proportion of indigenous forests in the upstream catchment.

Using random forest models, we demonstrated that the composition of bacterial communities could be used to correctly identify the catchment land use of the sample (as rural, urban or native forest) with 65% accuracy. However, this accuracy was primarily driven by the correct assignments of rural sites, which were correctly assigned 90% of the time (1897 out of 2100; Fig. 1b). Exotic forest samples were always incorrectly assigned as from either native forests or rural catchments (Fig. 1b).

**Fig. 2:**
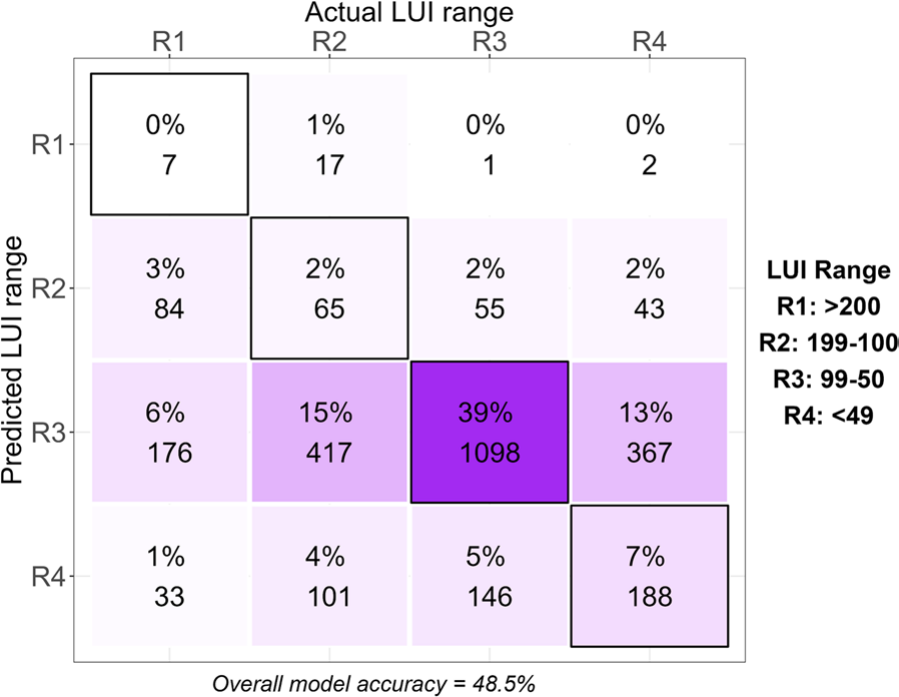
The number of correct and incorrect predictions of catchment land use index categories, based on 100 iterations of random forest classification using 183 stream bacterial ASVs representing the biological variation across disturbed samples. Black borders indicate correct classifications. Both proportions (percentages) and counts are given. Higher LUI values represent more degraded sites

Overall, we demonstrated that the disturbed sites could be assigned to their land use intensity category (LUI + 1) with 48.5% accuracy using 183 stream bacterial ASVs, representing the biological variation across samples (Fig. 2). Sites with a moderate LUI score (99–50, classed as ‘R3’ for the models) were assigned correctly 85% of the time, while those with the highest LUI score (> 200, ‘R1) were never assigned correctly (Fig. 2).

**Fig. 3:**
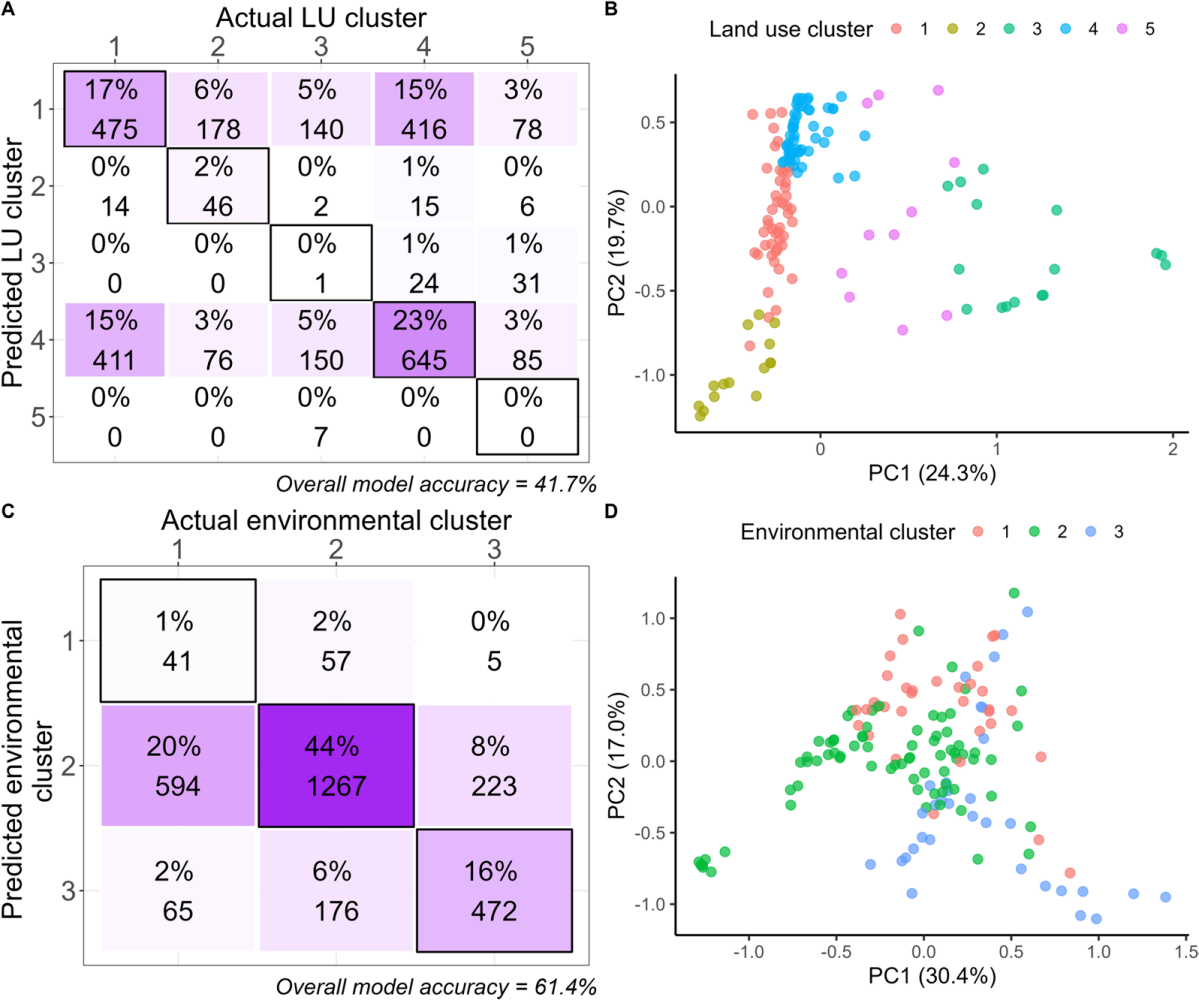
The number of correct and incorrect identifications of disturbed sites based on their **a** upstream catchment land use composition and **c** catchment-scale environmental data of the upstream catchments based on 100 iterations of random forest classifications of 183 stream bacterial ASVs, which represent the biological variation across disturbed samples. Black borders indicate correct classifications. Both proportions (percentages) and counts are given. PCA plots to the right show the underlying differences of sites in each cluster based on **b** land use characteristics and **c** environmental conditions. Each cluster can be further defined by the characteristics of the sites within those clusters, as in Tables S3–4

To determine if bacterial community characteristics could more accurately predict the percentage land use make-up of upstream catchments (comprising relative areas of the catchment under scrub, plantation forest, urban development, etc.), rather than their dominant land use pressure (as quantified via the land use index), disturbed sites were clustered into five groups based on the characteristic land uses in the upstream catchment (Fig. 3b, Table S3). Iterative use of random forest analyses showed that the land use clusters to which disturbed sites belonged could be correctly identified in 41.7% of cases using the subset of stream bacterial ASVs, which represent the biological variation across samples (Fig. 3a). Clusters one and four, which had the largest sample sizes (n = 47 and n = 53 respectively), were predicted with the greatest accuracy (52.8% and 58.6%) while the smaller clusters were poorly predicted, being more often wrong than right (Fig. 3a).

The disturbed sites could also be clustered into three groups based on the environmental data (Fig. 3d; Table S4). Analysis of random forest models confirmed that the environmental data clusters representing the disturbed sites could be correctly identified in 61.4% of cases, using the subset of stream bacterial ASVs, which represent the biological variation across samples (Fig. 3c) and 75.3% when the same approach was used to assess undisturbed sites (Fig. S5a). Despite clusters one and three having similar sample sizes, cluster 1 was almost always assigned incorrectly, while cluster 3 was correct 67% of the time (Fig. 3c).

**Fig. 4:**
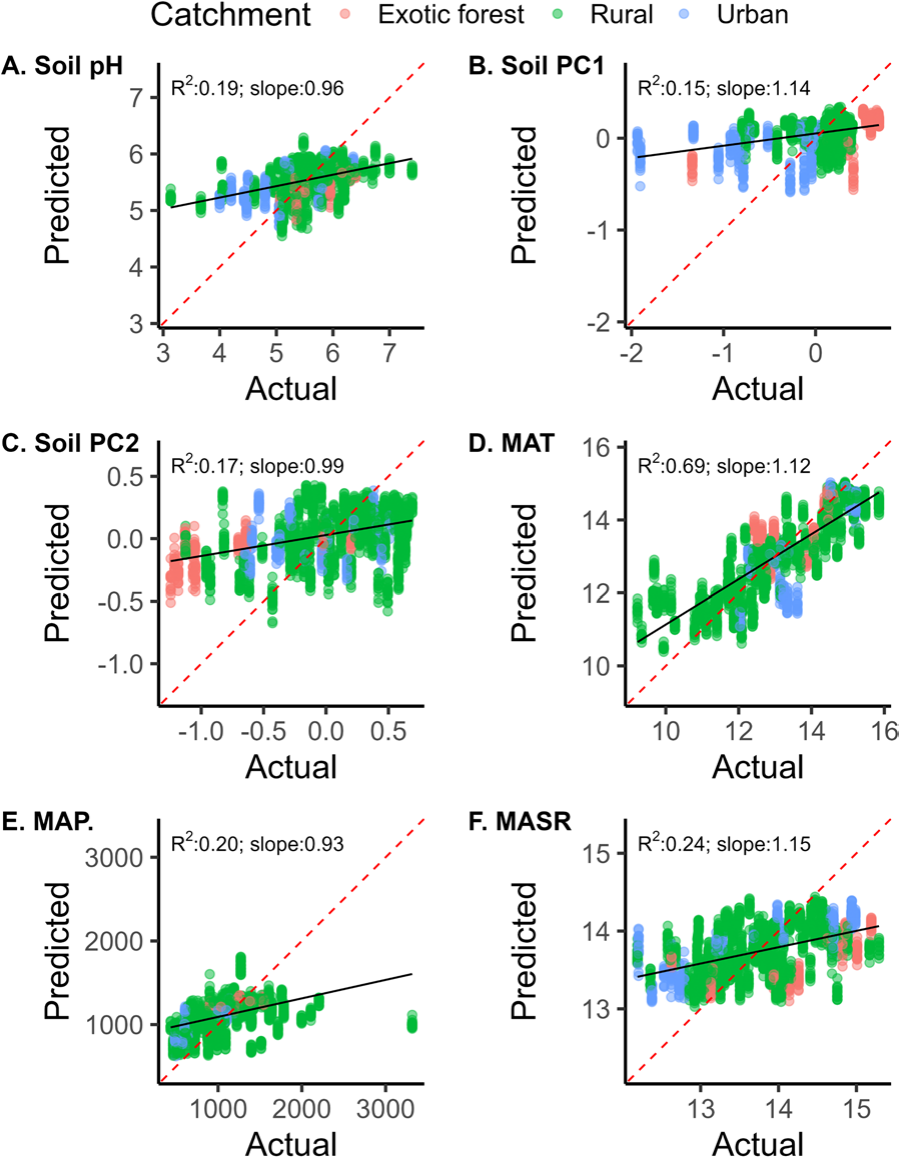
Accuracy of the random forest models in predicting **a** soil pH, soil physicochemical data PCA axes scores **b** one and **c** two, **d** mean annual temperature (°C), **e** mean annual precipitation (mm) and **f** mean annual solar radiation (mJ/m^2^/day). Dashed red lines indicate where points should fall for an exact prediction, while solid black lines represent the linear regression for each model’s predicted versus actual values. R^2^ and slope values are indicated in the upper left of each linear regression plot. Each plot contains predicted scores from 100 iterations of the random forest models, using different randomly selected subsets of data each time. Abbreviations are detailed in the supplementary material

We also assessed the ability of the stream bacterial ASVs, which captured the biological variation across samples, to predict individual environmental variables or the combined impact of soil physicochemical variables (PCA scores of the collective physicochemical data) for the disturbed dataset (Fig. 4; Fig. S6) and the undisturbed sites (Fig. S7). Regression analysis comparing the predicted to measured variables across 100 iterations of the random forest models mostly ranged from weak to moderate correlations (adjusted* R*^2^ from 0.02 to 0. 24) with one exception. This is when predicting the annual mean air temperature, where there was a strong correlation between the predicted and measured values (slope: 1.12; *R*^2^: 0.69; Fig. 4). Soil pH and mean annual solar radiation were the next most successful models (Fig. 4). Average log nitrogen concentration and percentage carbon in the catchment were not able to be predicted by bacterial community composition (Fig. S6). For the undisturbed sites, R^2^ values raged from 0.04 to 54; again, mean annual temperature was the most accurate model (Fig. S7). For all models, the regression analyses show a large variation for the same sample in different models, highlighting the importance of running the models on iterations of random data subsets (Fig. 4, Figs. S6–7).

**Fig. 5:**
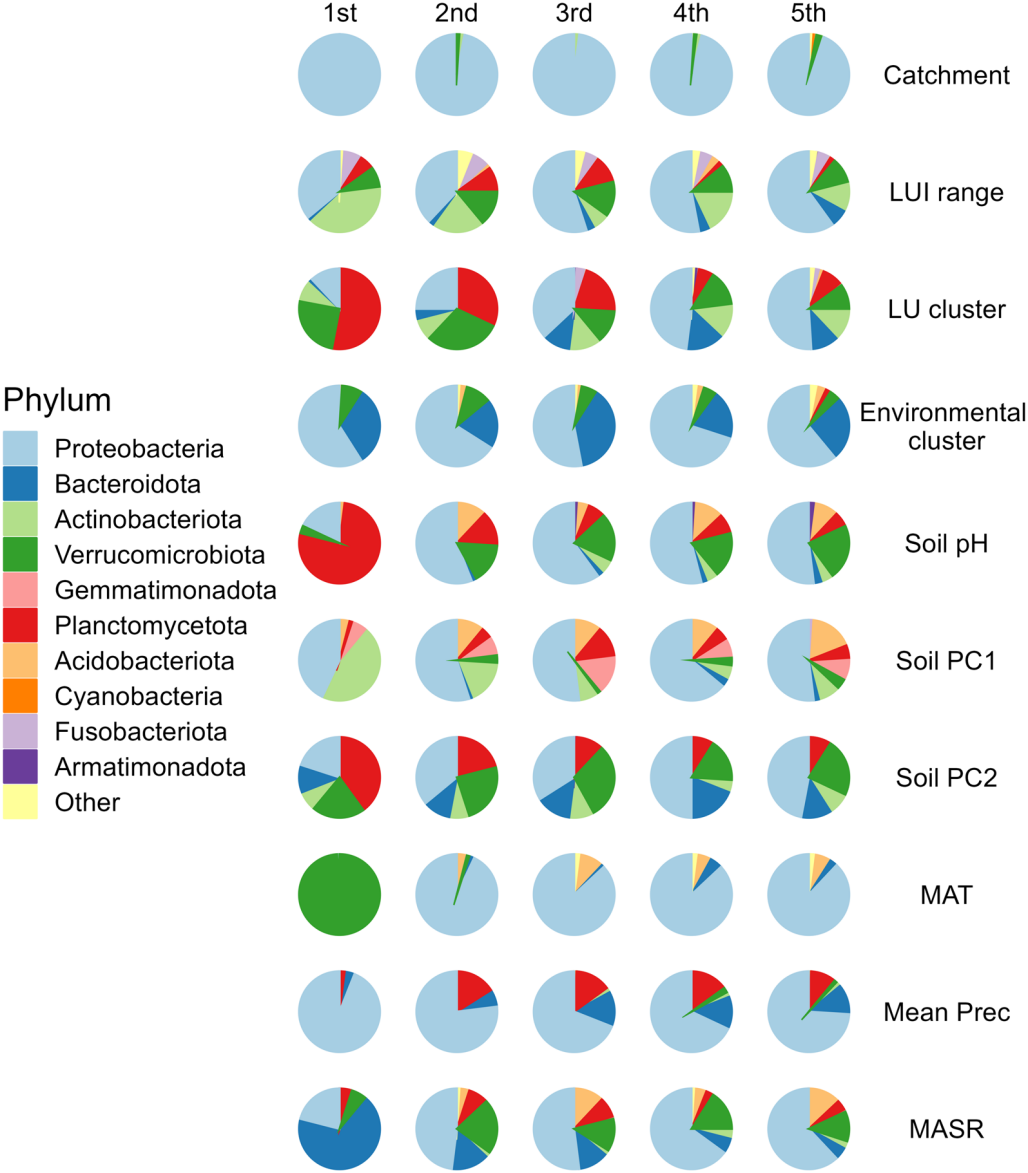
Phylum-level classification of the top five most important ASVs determined for each iteration of the random forest model. Columns represent the rank of ASV (1st is most important), and rows are the different models. Each pie shows the proportion of times ASVs classified as a particular phylum were ranked as important across the 100 iterations of the model. ASV importance was based on either the mean decrease accuracy associated with the ASV being excluded (for classification tree analyses—the first four models) or the % increase in mean squared error (for regression tree analysis—the last six models). Abbreviations are detailed in the supplementary material

Proteobacteria, Bacteroidota and Actinobacteriota were most commonly in the top five most important ASVs for the accuracy of each model (Fig. 5). For the ‘catchment’ model (Fig. 1b), the top five most important ASVs were almost always Proteobacteria. In contrast, most other models had various phyla that were considered the five most important across the different iterations (Fig. 5). For the MAT model (Fig. 4d), the first most important ASV was almost always a Verrucomicrobiota, while the remaining four most important ASVs were most often Proteobacteria (Fig. 5). For some models, including the land use clusters (Fig. 3a), soil pH (Fig. 4a) and soil PC2 scores (Fig. 4c), Planctomycetota were also commonly among the five most important ASVs.

## Discussion

To date, relatively few studies have attempted to monitor microbial DNA as a routine tool to monitor freshwater catchments [24, 27, 43], with assessments of fish and macroinvertebrate communities remaining more common [15]. Capitalising on decades of research facilitating the establishment of macroinvertebrate indices of community health, early efforts used regression analyses to identify microbial community attributes associated with different macroinvertebrate community compositions (i.e., macroinvertebrate community types indicative or poor- to high-quality stream environments, Lau et al. [27])*.* However, such approaches are then limited by the quality of the macroinvertebrate indices that they attempt to emulate. Avoiding such limitations, we used bacterial DNA data alone to confirm that bacterial community composition was clearly differentiated by land use type and physicochemical characteristics in the upstream catchments of 204 New Zealand streams. Exploring the diagnostic potential of stream bacterial communities as an indicator of anthropogenic impact, we have shown that bacterial community composition could be used to correctly identify site land use assignments with 65% accuracy. Key catchment soil physicochemical traits could be identified with up to 75% accuracy for sites dominated by an undisturbed catchment. These results provide crucial information for ecosystem monitoring initiatives using stream bacterial communities.

We completed a nationwide survey of stream biofilm bacterial community distribution across four types of catchment land use across New Zealand. Whilst the composition and distribution of stream bacterial communities varied regionally [28], bacterial community structure was nevertheless significantly impacted by the catchment land use and associated physicochemical attributes (Fig. 1a). We successfully derived indicator bacterial taxa from our initial stream bacterial dataset by identifying priority ‘indicator ASVs’ with maximal ability to differentiate among groups of data determined by our clustering technique. From our bacterial community data, we investigated the ability to correctly infer both the type and extent of anthropogenic impact in upstream catchments distributed over a ~ 1000-km latitudinal gradient, observing a significant overlap of data representing native and rural sites. We could not obtain satisfactory predictions using models constructed from crude assignments of catchment types. This is understandable since some streams designated as rural, dominated by grassland in the upstream catchment, possessed LUI values similar to some native forest sites, which nevertheless contained a small portion of urban area in the upstream catchment (i.e., having LUIs of about 75). Thus, dominant upstream catchment type, or LUI scores, might not provide the best measures of land use impact at the point of sampling. Increasing the sampling size of some more underrepresented catchment types might also improve the models. Indeed, the lack of site data in the training data set from under exotic forest prevented attempts to correctly assign samples to this land use (Fig. 1b).

Deterioration in stream health has drawn concerns worldwide [37–39, 47]. Here, we used LUI scores to quantify various land use factors (reflecting urban, pasture and cropland—including exotic forest). However, our random forest approach incorrectly interpreted LUI groupings. The crude land use assignments devised by Neale et al. [40] provided better model outputs than when using LUI scores as input data. The inclusion of more detailed data (i.e., incorporating upstream percentage land use data in addition to LUI scores did not improve the ability of our random forest approach to correctly assign disturbed sites to their characteristic land use attributes. This suggests that perhaps it is the most dominant land use cover that most strongly impacts the stream biofilm communities, rather than a combination of all land uses in the catchment.

While improvements and refinements are needed, the ability of our models to predict sample catchment land use characteristics more often than by chance alone supported the idea that bacterial community data may further be used to estimate other stream catchment parameters, including those related to water quality. Mean annual air temperature was the most accurately predicted variable from random forest regression models. Temperature influences physiological processes, possibly leading to successional changes in bacterial communities and impacting trophic interactions [25, 54]. Hence, our model approach offers an alternative strategy to quantify the impact of land use and climate change on bacterial communities. Using catchment physicochemical parameters as model input data, we determined that analysis of bacterial community data could be used to identify some groups of physicochemical parameters, particularly pH correctly, but also combined measures of catchment status (i.e. using data from principal components axis one, combining various catchment land use and physicochemical data). Such factors can greatly impact cyanobacterial and proteobacterial communities [4, 22, 60]. Indeed, Proteobacteria were most often identified as important taxa for improving the accuracy of the models. Average log nitrogen concentrations in the water and mean carbon percentage in the surface soil of the upstream catchment showed the weakest relationship between actual and predicted values. Other studies reveal relationships between physicochemical conditions and microbial biomass, abundances and rates of key processes such as CO_2_ production and biological oxygen demand [26, 31, 46]. Thus, it remains likely that different types of microbial data may better predict different stream parameters and that combining microbial biomass, abundance, composition, and process data may strengthen future models aiming to predict stream catchment status better.

The variation in accuracy with which different measured variables could be predicted could be explained by the fact that many factors independent of land use drive stream bacterial community differences. Our studies, using a similar dataset, confirm the presence of spatial gradients in stream bacterial community composition, including both latitudinal and elevational gradients in community similarity [28], suggesting a potential role for dispersal limitation in shaping microbial community structure. Indeed, we previously confirmed increases in bacterial taxonomic richness in stream biofilm bacterial communities sampled further north (closer to the equator) within New Zealand and a decline in the average latitude range of taxa (in this case, 97% OTUs) by 28 km for every 100 km north travelled [29]. While spatial differences in bacterial community composition are thought to be primarily determined by niche-based processes (i.e. species sorting) rather than by neutral processes (i.e. community similarity driven by spatially limited dispersion [28, 56]), such findings highlight the importance of including these data to further improve the efficacy of the random forest approach, allowing spatial and climate-related trends may be accounted for. Likewise, incorporating a temporal element into data gathering could improve the random forest models, or at least would be important for validating the extent to which models built on data collected at one time can be applied to infer information based on data collected at a different time.

The diagnostic potential of bacterial communities as indicators, especially in combination with machine learning approaches, has significant appeal for freshwater research [14]. Including bacterial community data in bioindicator models is achievable mainly due to the greater availability of high throughput sequencing, bioinformatics and statistical approaches that facilitate rapid assessment and annotation of bacterial community attributes, for example, 16S rRNA genes [49]. Incorporating metagenomics and transcriptomics techniques could further enhance our ability to describe environmental conditions and changes in land use [5, 21, 48]. Indeed, models could be adapted to target different microbial processes via the selection of functional gene sets, such as methyl coenzyme M reductase (*mcr*) genes targeting methanogens, membrane-bound particulate (*pMMO*) encoding methane monooxygenase enzymes used for methanotrophy, or the *ars* genes conferring resistance to arsenate, arsenite and antimonite. Though further work would be required to validate the effectiveness of models based on functional data and determine the impact of functional redundancy on such models.

## Conclusion

Recognition of the impact of land use differences on stream bacterial community abundance, composition and function suggests that bacterial community data may be used to monitor certain impacts of land use and changing environmental conditions on freshwater systems [1, 33, 52]. Our analysis framework emphasises a biologically relevant approach to understanding and identifying differences in bacterial community composition by explicitly considering environmental constraints. Overall, we recommend machine learning approaches, perhaps incorporating functional approaches to characterise the potential of stream bacterial communities as an alternative indicator of diverse catchment attributes.

### Supplementary Information

Supplementary Material 1.

## Data Availability

All sequence and associated data are provided in NCBI Sequence Read Archive under project accession number PRJNA328535.
