# Supplementary material for: Exploring freshwater stream bacterial communities as indicators of land use intensity
Source: Environ Microbiome. 2024 Jul 8;19:45. doi: 10.1186/s40793-024-00588-z (PMC11232138; doi:10.1186/s40793-024-00588-z)
Supplement: Supplementary file 1 — Supplementary Material 1. [file 40793_2024_588_MOESM1_ESM.docx]

**Supplementary Materials**

**List of Abbreviations:**

*Ave. LogN* – annual average concentration of total nitrogen in the stream water (log ppb), extracted from the Freshwater Environments of New Zealand (FENZ) database (Leathwick et al., 2010).

*Elevation* – Elevation above sea level, in metres.

*Land use index* – calculated using the equation ‘(4 x urban + 2 x crop + 1 x pasture) + 1’, where land uses represent percentage coverage within each catchment.

*MASR* – Mean annual solar radiation at the sampling location (mJ/m^2^/day), extracted from the National Institute of Water and Atmospheric Research, New Zealand (Wratt et al., 2006).

*Mean C Percent* – Mean percent carbon in the catchment soil at 0.2 m depth, extracted from the New Zealand Land Information (NZLRI) database (Leathwick et al., 2010).

*Mid pH* – Median soil pH, extracted from the New Zealand Land Resource Information System (NZLRI) database (Newsome et al., 2000).

*PC1*- First axis score of principal components analysis of all physicochemical data.

*PC2* – Second axis score of principal components analysis of all physicochemical data.

*Plantation Forest* - Percent of catchment under plantation forestry, as extracted from the Land Cover Database 2 (MfE, 2004).

*Prec. Mean –* Annual mean precipitation (mm), provided by the National Institute of Water and Atmospheric Research (NIWA), New Zealand.

*Prec. SD* – Variation in precipitation (standard deviation) comparing monthly precipitation data), provided by NIWA.

*Urban Areas* – Percent of catchment under urban land use, as extracted from the Land Cover Database 2 (MfE, 2004).

Figure S1. A total of 204 stream sites were sampled for bacterial community analysis from within seven regions administrated by the following councils: Auckland Council (AK), Environment Waikato (WK), Hawkes Bay (HB), Horizons (Manawatu–Wanganui) (HR), Greater Wellington (WG), Tasman (TS) and Environment Canterbury (CB). Sampling locations were located from 36°20′40.24″S to 44°54′37.81″S (latitude) and 169°35′03.62″E to 177°35′03.62″E (longitude).

Table S1. Land use index (LUI) scores for all sites.

| Site | Region | Catchment | *landuse_index+1 |
| --- | --- | --- | --- |
| AKOAK | AK | Urban | 390.41 |
| HBRHP | HB | Urban | 388.93 |
| CBDUD | CB | Urban | 384.84 |
| TSBOR | TS | Exotic Forest | 306.06 |
| AKOTH | AK | Urban | 299.05 |
| AKEL | AK | Urban | 270.00 |
| AKCH | AK | Urban | 268.40 |
| CBWMR | CB | Urban | 242.83 |
| AKO | AK | Urban | 241.43 |
| WGWAI | WG | Urban | 237.58 |
| AKPHN | AK | Urban | 235.75 |
| AKCB | AK | Urban | 233.85 |
| CBSTX | CB | Rural | 227.13 |
| TSJLB | TS | Urban | 217.62 |
| WGMGP | WG | Rural | 213.70 |
| TSTAS | TS | Rural | 202.26 |
| WGKAR | WG | Urban | 198.07 |
| AKLG | AK | Urban | 189.91 |
| CBTNK | CB | Rural | 182.31 |
| AKLU | AK | Urban | 180.69 |
| WGPOR | WG | Rural | 158.55 |
| WKPCK | WK | Rural | 151.54 |
| CBKP | CB | Rural | 147.33 |
| HRHOK | HR | Rural | 141.63 |
| TSRES | TS | Urban | 140.63 |
| AKMU | AK | Rural | 138.35 |
| AKEM | AK | Urban | 131.25 |
| CBOHP | CB | Rural | 126.26 |
| AKOTR | AK | Urban | 119.85 |
| TSJLW | TS | Urban | 118.38 |
| AKNK | AK | Rural | 118.32 |
| CBAKR | CB | Rural | 118.32 |
| AKPB | AK | Urban | 118.16 |
| HBMNT | HB | Urban | 117.91 |
| CBCMD | CB | Rural | 117.32 |
| HBHER | HB | Urban | 110.66 |
| WGKWH | WG | Urban | 108.79 |
| CBBOG | CB | Rural | 106.87 |
| AKDC | AK | Rural | 105.60 |
| CBSKL | CB | Rural | 105.32 |
| CBGLN | CB | Rural | 104.58 |
| HBPGH | HB | Rural | 103.44 |
| CBLYL | CB | Rural | 103.27 |
| AKWG | AK | Rural | 103.18 |
| WKMTA | WK | Rural | 101 |
| WKNK | WK | Rural | 100.35 |
| WKMSB | WK | Rural | 99.23 |
| WGPKW | WG | Rural | 99.00 |
| CBWK | CB | Rural | 96.93 |
| CBDRY | CB | Rural | 96.18 |
| CBABT | CB | Native Forest | 95.78 |
| HBMNK | HB | Urban | 95.49 |
| AKK | AK | Rural | 92.21 |
| AKPU | AK | Rural | 91.80 |
| HRMWB | HR | Rural | 90.92 |
| AKPP | AK | Rural | 90.60 |
| HBPUH | HB | Rural | 90.50 |
| CBCL | CB | Rural | 90.50 |
| CBSR | CB | Rural | 90.21 |
| HBTKP | HB | Rural | 89.97 |
| HRMW | HR | Rural | 89.37 |
| AKMK | AK | Native Forest | 87.86 |
| HBMGK | HB | Rural | 87.74 |
| HRORO | HR | Rural | 87.56 |
| HRMPT | HR | Rural | 85.44 |
| AKD | AK | Rural | 85.20 |
| TSRED | TS | Exotic Forest | 84.89 |
| AKM | AK | Rural | 84.59 |
| HBPOR | HB | Rural | 84.37 |
| HBTUK | HB | Rural | 84.06 |
| HBTRB | HB | Rural | 83.90 |
| HRMWW | HR | Rural | 83.62 |
| HRTH | HR | Rural | 83.05 |
| HRBR | HR | Rural | 82.56 |
| CBWPR | CB | Rural | 82.18 |
| HRMUG | HR | Rural | 81.60 |
| HRMAN | HR | Native Forest | 81.44 |
| HBTSH | HB | Rural | 81.39 |
| HRTIR | HR | Rural | 81.25 |
| HRMO | HR | Rural | 81.09 |
| HBMAN | HB | Rural | 80.49 |
| WGTR | WG | Rural | 78.38 |
| CBOPH | CB | Rural | 77.17 |
| AKLV | AK | Rural | 76.30 |
| CBPAW | CB | Rural | 75.48 |
| AKOT | AK | Rural | 74.66 |
| WKHW | WK | Rural | 73.88 |
| WKWGR | WK | Native Forest | 72.47 |
| CBMB | CB | Rural | 71.54 |
| WKWK | WK | Native Forest | 71.34 |
| CBKTN | CB | Rural | 70.14 |
| WKWN | WK | Native Forest | 69.28 |
| HBHAN | HB | Rural | 68.49 |
| CBBAL | CB | Rural | 66.68 |
| AKART | AK | Rural | 66.42 |
| CBSEL | CB | Rural | 66.17 |
| AKVU | AK | Rural | 66.02 |
| WGAW | WG | Rural | 65.66 |
| CBHAL | CB | Rural | 65.59 |
| CBPRS | CB | Rural | 64.52 |
| HRTS | HR | Rural | 64.49 |
| AKSH | AK | Rural | 62.90 |
| HRHT | HR | Rural | 62.68 |
| CBQLB | CB | Rural | 62.29 |
| HROAL | HR | Native Forest | 61.40 |
| HBMNR | HB | Rural | 59.16 |
| HRMWP | HR | Rural | 58.79 |
| HRKH | HR | Rural | 57.00 |
| WKPRN | WK | Native Forest | 56.04 |
| WGMTR | WG | Rural | 55.82 |
| HBESK | HB | Rural | 55.61 |
| WGTRC | WG | Rural | 54.03 |
| WKMNR | WK | Native Forest | 53.78 |
| CBOPU | CB | Rural | 53.37 |
| AKDCP | AK | Rural | 53.01 |
| CBPAH | CB | Rural | 50.96 |
| AKWB | AK | Native Forest | 50.59 |
| CBIRC | CB | Rural | 50.53 |
| CBOMR | CB | Rural | 49.90 |
| AKARM | AK | Rural | 49.89 |
| HBTP | HB | Rural | 49.78 |
| CBASH | CB | Rural | 49.42 |
| WGHOR | WG | Rural | 49.03 |
| WKWMU | WK | Native Forest | 48.54 |
| AKAR | AK | Rural | 48.43 |
| HRWG | HR | Native Forest | 47.86 |
| AKOK | AK | Rural | 46.94 |
| WKOMW | WK | Native Forest | 46.70 |
| AKSY | AK | Rural | 45.70 |
| HBMAK | HB | Rural | 44.78 |
| AKMTK | AK | Rural | 44.50 |
| TSJLH | TS | Exotic Forest | 44.24 |
| HRRMW | HR | Rural | 43.88 |
| HBWP | HB | Rural | 40.98 |
| WKOAM | WK | Native Forest | 40.96 |
| HBTKT | HB | Rural | 40.92 |
| WKTRP | WK | Native Forest | 38.51 |
| WGMAN | WG | Native Forest | 38.24 |
| WGWHB | WG | Native Forest | 37.39 |
| HBRUA | HB | Rural | 37.25 |
| AKTRN | AK | Native Forest | 36.42 |
| CBAG | CB | Native Forest | 35.42 |
| AKAWN | AK | Rural | 32.78 |
| HRRG | HR | Native Forest | 32.52 |
| HRWC | HR | Native Forest | 30.14 |
| WGHUT | WG | Native Forest | 30.05 |
| AKOP | AK | Rural | 26.98 |
| AKAU | AK | Rural | 26.69 |
| AKPH | AK | Native Forest | 25.55 |
| TSRM | TS | Exotic Forest | 23.92 |
| AKKT | AK | Native Forest | 22.71 |
| CBFRK | CB | Native Forest | 21.63 |
| HBMKD | HB | Native Forest | 19.05 |
| AKKKP | AK | Native Forest | 18.94 |
| CBHRN | CB | Native Forest | 18.87 |
| HRTK | HR | Native Forest | 18.65 |
| AKWH | AK | Native Forest | 18.36 |
| CBEAL | CB | Rural | 17.54 |
| WKOKU | WK | Rural | 16.11 |
| HBMRP | HB | Native Forest | 15.44 |
| HBMWF | HB | Native Forest | 13.95 |
| CBSPR | CB | Rural | 13.51 |
| HRPHP | HR | Native Forest | 13.49 |
| HROAG | HR | Native Forest | 13.07 |
| WKKRK | WK | Native Forest | 12.75 |
| CBKOW | CB | Native Forest | 12.40 |
| AKRH | AK | Exotic Forest | 11.72 |
| AKHKH | AK | Exotic Forest | 9.81 |
| WKTRK | WK | Native Forest | 9.79 |
| HRMD | HR | Native Forest | 9.59 |
| WKWTT | WK | Rural | 8.81 |
| WKPIR | WK | Native Forest | 7.12 |
| WGHTM | WG | Native Forest | 7.07 |
| WKPHE | WK | Native Forest | 6.33 |
| HBWKR | HB | Rural | 6.29 |
| AKNKU | AK | Native Forest | 4.92 |
| WKTRU | WK | Native Forest | 3.99 |
| WGWKN | WG | Native Forest | 3.27 |
| CBBSH | CB | Native Forest | 2.50 |
| HBMKM | HB | Native Forest | 2.24 |
| HBMKU | HB | Native Forest | 1.65 |
| WGWG | WG | Native Forest | 1.50 |
| AKMW | AK | Native Forest | 1.44 |
| HBTIW | HB | Exotic Forest | 1.26 |
| HBANI | HB | Native Forest | 1.05 |
| WKMTU | WK | Native Forest | 1.02 |
| AKWS | AK | Rural | 1 |
| AKAWD | AK | Exotic Forest | 1 |
| AKMH | AK | Exotic Forest | 1 |
| AKOB | AK | Exotic Forest | 1 |
| AKSP | AK | Exotic Forest | 1 |
| AKDCB | AK | Rural | 1 |
| AKEU | AK | Urban | 1 |
| AKWK | AK | Native Forest | 1 |
| AKCS | AK | Native Forest | 1 |
| AKKN | AK | Native Forest | 1 |
| AKMA | AK | Native Forest | 1 |
| AKML | AK | Native Forest | 1 |
| AKOR | AK | Native Forest | 1 |
| AKOTN | AK | Native Forest | 1 |
| WKAHR | WK | Native Forest | 1 |
| WKKAU | WK | Native Forest | 1 |
| WKWKK | WK | Native Forest | 1 |
| WGBC | WG | Native Forest | 1 |

*Land use index (LUI) values were calculated for each site using the equation ‘4 x urban + 2 x crop + 1 x pasture’ and adding +1 to each site to eliminate the presence of any zero values for equal and comparable interpretation. In this index, adapted from Collier (2008), the terms ‘urban’, ‘crop’ and ‘pasture’ refer to the percentage of those land uses in the upstream catchment. Higher LUI values are then expected to represent more degraded catchments.

Table S2. Summary of the subsets of data used in each model. For each subset type (‘all’, ‘disturbed’ or ‘undisturbed’), indicator ASVs were obtained by separating the ASV by performing Ward’s minimum clustering on Bray-Curtis dissimilarity matrices of the bacterial community data. Indicator analysis was then used to select the ASVs representing the variation for each cluster; these ASVs were then used in the random forest analyses. For the land use or environmental data clusters, Ward’s minimum clustering on explanatory variables was done to assign clusters. For each model, 80% of the sites were used to train the model, and the remaining 20% were used to test the model; sites were randomly selected using stratified sampling.

| Model | Subset used | # of sites | # of ASV clusters | # of indicator ASVs | Variables used for clustering | # of clusters | Variable used for stratified subsampling | Model type |
| --- | --- | --- | --- | --- | --- | --- | --- | --- |
| Catchment type (Figure 1b) | All sites | 204 | 5 | 226 |  |  | Catchment type | Classification tree |
| LUI range (Figure 2) | Disturbed sites: exotic forests, rural or urban | 142 | 3 | 183 |  |  | LUI range | Classification tree |
| Land use clusters  (Figure 3a) | Disturbed sites: exotic forests, rural or urban | 142 | 3 | 183 | LUI, proportion of bare ground,  plantation forest, indigenous forest, grassland, cropland, scrub, urban areas and wetlands in the catchment. | 5 | Land use cluster | Classification tree |
| Environmental clusters  (Figure 3c) | Disturbed sites: exotic forests, rural or urban | 142 | 3 | 183 | Mean precipitation, variation in precipitation, average stream N, mean soil C, median soil pH, MASR, MAT and elevation. | 3 | Environmental cluster | Classification tree |
| Environmental variables (Figure 4) | Disturbed sites: exotic forests, rural or urban | 142 | 3 | 183 |  |  | Catchment type | Regression |
| Environmental clusters – undisturbed sites  (Figure S5a) | Undisturbed sites: native forests | 62 | 5 | 397 | Mean precipitation, variation in precipitation, average stream N, mean soil C, median soil pH, MASR, MAT and elevation. | 2 | Environmental cluster | Classification tree |
| Environmental variables – undisturbed sites  (Figure S7) | Undisturbed sites: native forests | 62 | 5 | 397 |  |  | Catchment type | Regression |


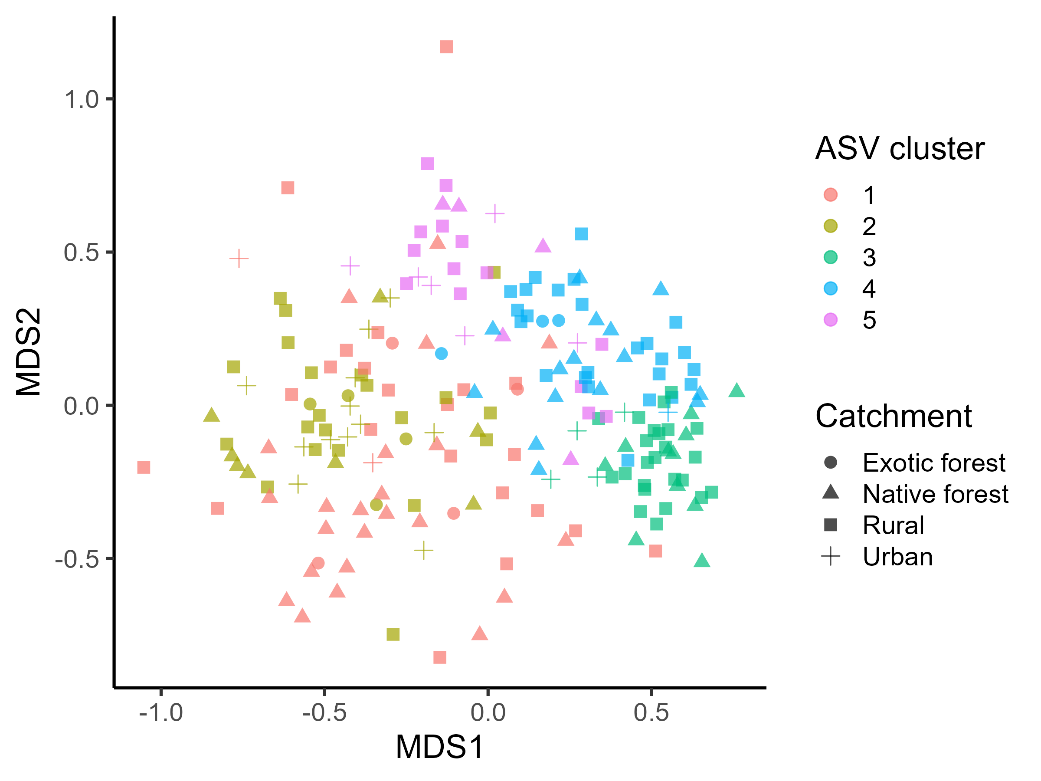


Fig S2: Underlying differences (Bray Curtis dissimilarity) in bacterial community across all stream biofilm samples collected from urban, rural, exotic– or native-forest catchments. Points are coloured according to the ASV cluster each site belongs to, as determined using Ward’s method for hierarchical clustering. Shapes indicate the catchment type the site belongs to.

**
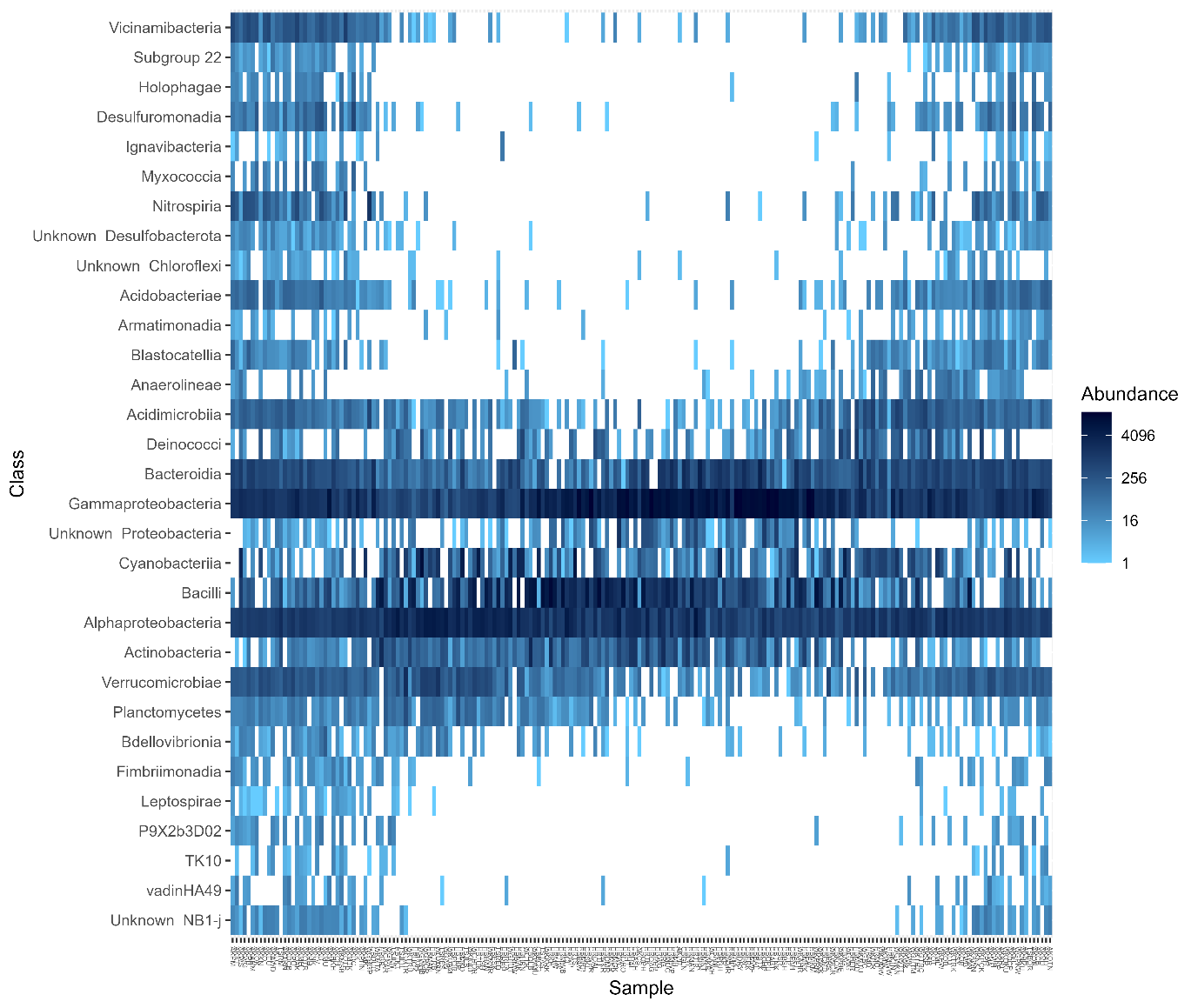
**

Fig S3. Heat map showing variation of the 226 ASVs, which were selected as representative of the biological variation across samples, grouped into Class level classifications. Ordination (NMDS of Bray-Curtis distances) was used to order samples. Each row represents a specific bacterial Class and each column a different stream sample. The colour gradient represents the relative abundance of each Order from low abundance (light blue) to high abundance (dark blue); white indicates an abundance of 0.


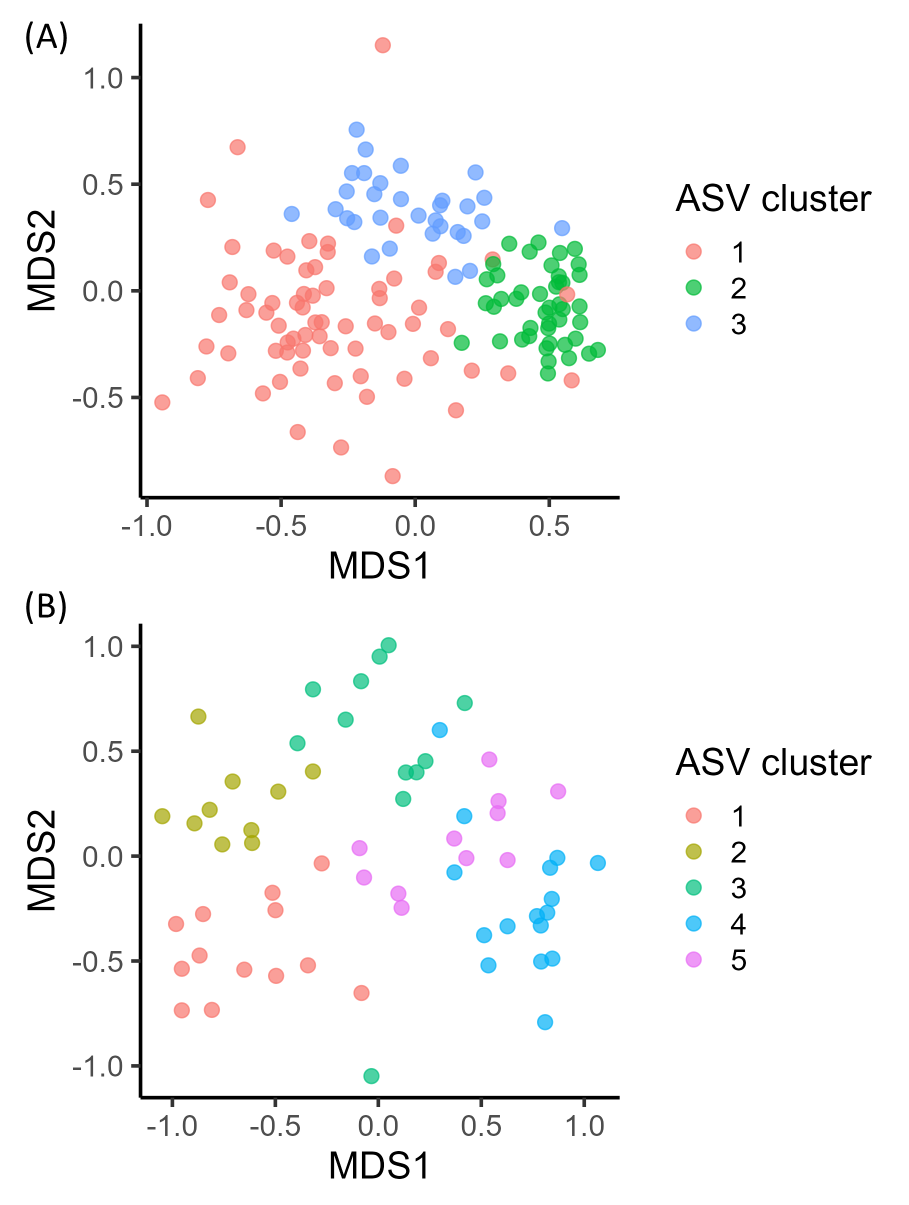


Figure S4: Underlying differences (Bray Curtis dissimilarity) in bacterial communities across the stream biofilm samples collected from (A) disturbed catchments (urban, rural or exotic forests) and (B) undisturbed catchments (native-forest). Points are coloured according to the ASV cluster each site belongs to, as determined using Ward’s method for hierarchical clustering.

Table S3. Differences in land use index (LUI) or land use cover (as a percentage of catchment) are associated with the five land use clusters (Figure 3a). Post-hoc tests revealed significantly different data, represented by different letters (Dunn’s P < 0.05), whereas the same letters were assigned to significantly similar clusters.

| **Land use variable** | **Cluster 1**  **n = 47** | **Cluster 2**  **n = 15** | **Cluster 3**  **n = 16** | **Cluster 4**  **n = 53** | **Cluster 5**  **n = 11** |
| --- | --- | --- | --- | --- | --- |
| LUI | 53.9^a^ | 8.6^a^ | 272.2^b^ | 96.9^c^ | 154.4^b c^ |
| Bare ground | 1.3^ab^ | 16.7^a^ | 0.05^c^ | 0.3^abc^ | 0.09^bc^ |
| Plantation forest | 8.8^a^ | 33.9^a^ | 3.1^b^ | 4.2^ab^ | 10.2^ab^ |
| Indigenous forest | 19.9^a^ | 39.0^a^ | 13.5^ab^ | 5.05^b^ | 10.1^ab^ |
| Grassland | 57.7^a^ | 5.3^b^ | 16.1^b^ | 82.6^c^ | 28.4^ab^ |
| Cropland | 0.4^ab^ | 0.0^b^ | 1.3^abc^ | 3.6^c^ | 20.6^ac^ |
| Scrub | 11.0^a^ | 3.6^b^ | 2.5^c^ | 2.3^abc^ | 9.5^abc^ |
| Urban areas | 0.5^a^ | 0.6^a^ | 63.1^b^ | 1.7^ac^ | 21.0^bc^ |
| Wetlands | 0.5^a^ | 0.9^b^ | 0.3^b^ | 0.3^a^ | 0.2^ab^ |

Table S4. Differences in environmental variables associated with the three environmental clusters (Figure 3c). Post-hoc tests revealed significantly different data, represented by different letters (Dunn’s P < 0.05), whereas the same letters were assigned to significantly similar clusters.

| **Environmental variable** | **Cluster 1**  **n = 34** | **Cluster 2**  **n = 75** | **Cluster 3**  **n = 33** |
| --- | --- | --- | --- |
| Mean precipitation (mm) | 1577^a^ | 1084^b^ | 630^c^ |
| Variation in precipitation (SD, mm) | 177^a^ | 43^b^ | 74^ab^ |
| Average stream N (log ppb) | 0.08^a^ | 0.07^a^ | 0.10^a^ |
| Mean soil C (%) | 19.9^a^ | 16.2^b^ | 19.0^b^ |
| Median soil pH | 5.6^ab^ | 4.9^a^ | 5.7^b^ |
| MASR (mJ/m^2^/day) | 13.7^ab^ | 13.5^a^ | 14.0^b^ |
| MAT (°C) | 13.4^a^ | 13.7^a^ | 11.6^b^ |
| Elevation (masl) | 137.6^a^ | 57.1^b^ | 136.1^b^ |


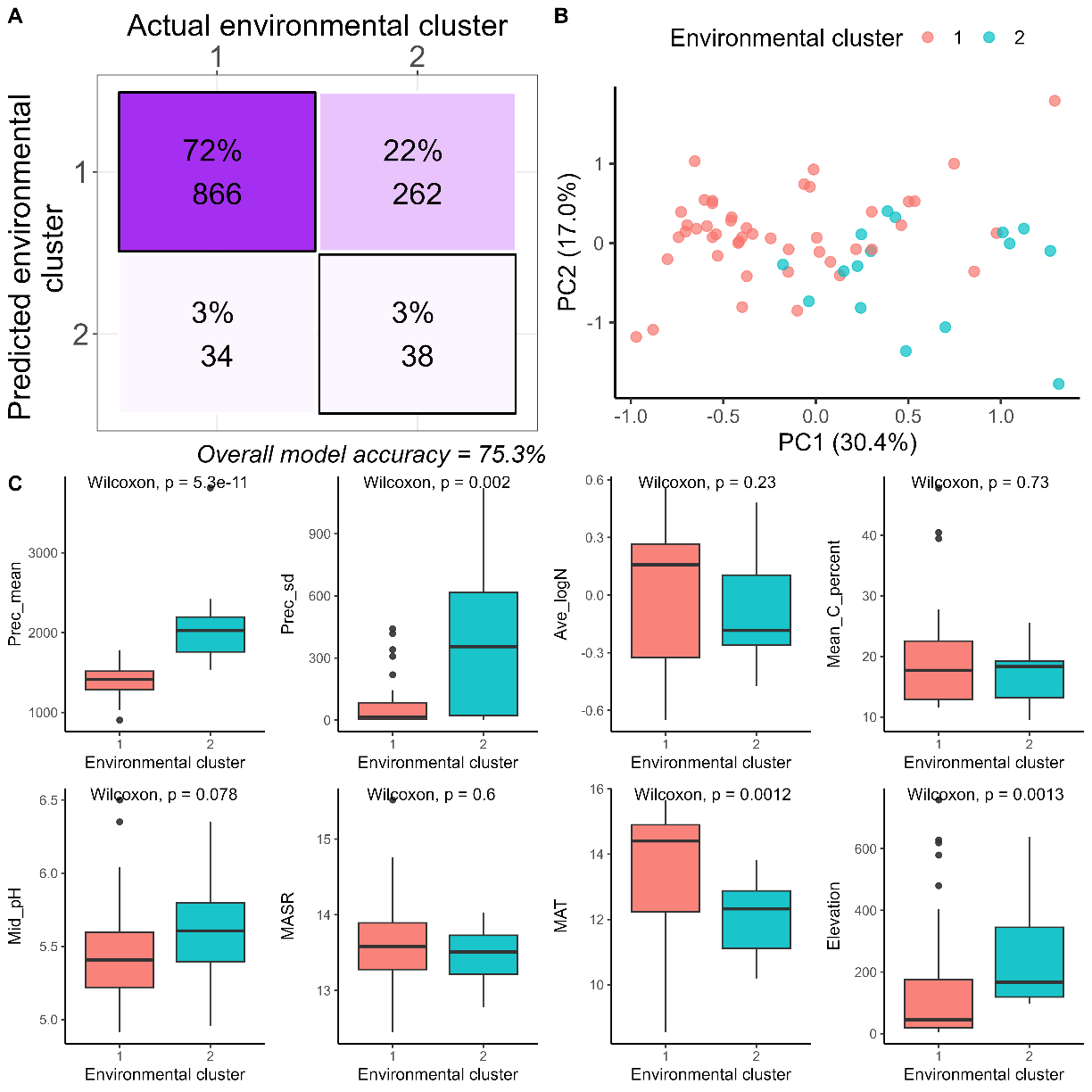


Figure S5 (A) The number of correct and incorrect identifications of the ‘environmental cluster’ of undisturbed sites based on 100 iterations of random forest classifications of 397 stream bacterial ASVs, which represent the biological variation across the undisturbed samples. Black borders indicate correct classifications. Both proportions (percentages) and counts are given. (B) PCA plots show the underlying differences of sites in each cluster. (C) The characteristics of sites in each cluster are based on the catchment-scale environmental data of the upstream catchments (which was used for clustering).


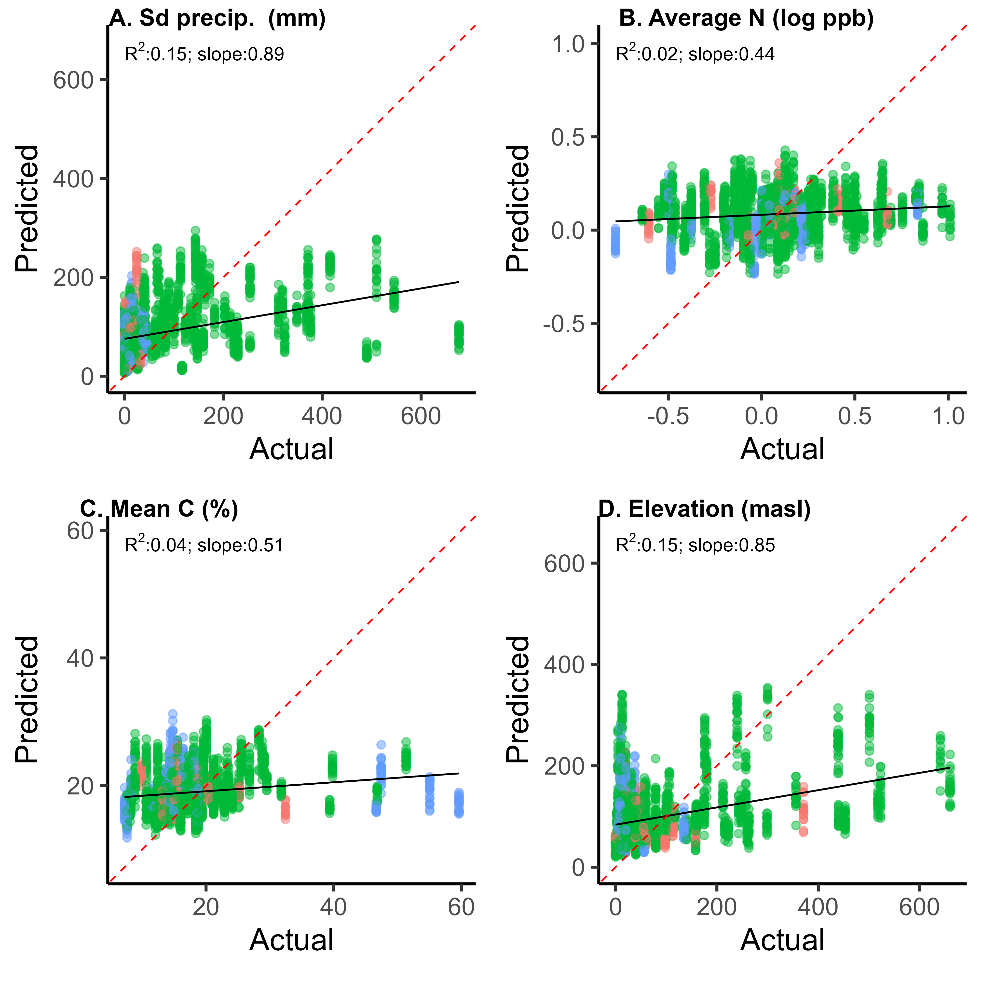


Figure S6. Accuracy of the random forest models in predicting (A) variation (standard deviation) in annual precipitation, (B) annual average concentration of total nitrogen in the stream water (log ppb), (C) mean percent carbon in the catchment soil at 0.2 m depth and (D) Elevation. Dashed red lines indicate where points should fall for an exact prediction, while solid black lines represent the linear regression for each model’s predicted versus actual values. R2 and slope values are indicated in the upper left of each linear regression plot. Each plot contains predicted scores from 100 iterations of random forest models, using different randomly selected subsets of data each time.


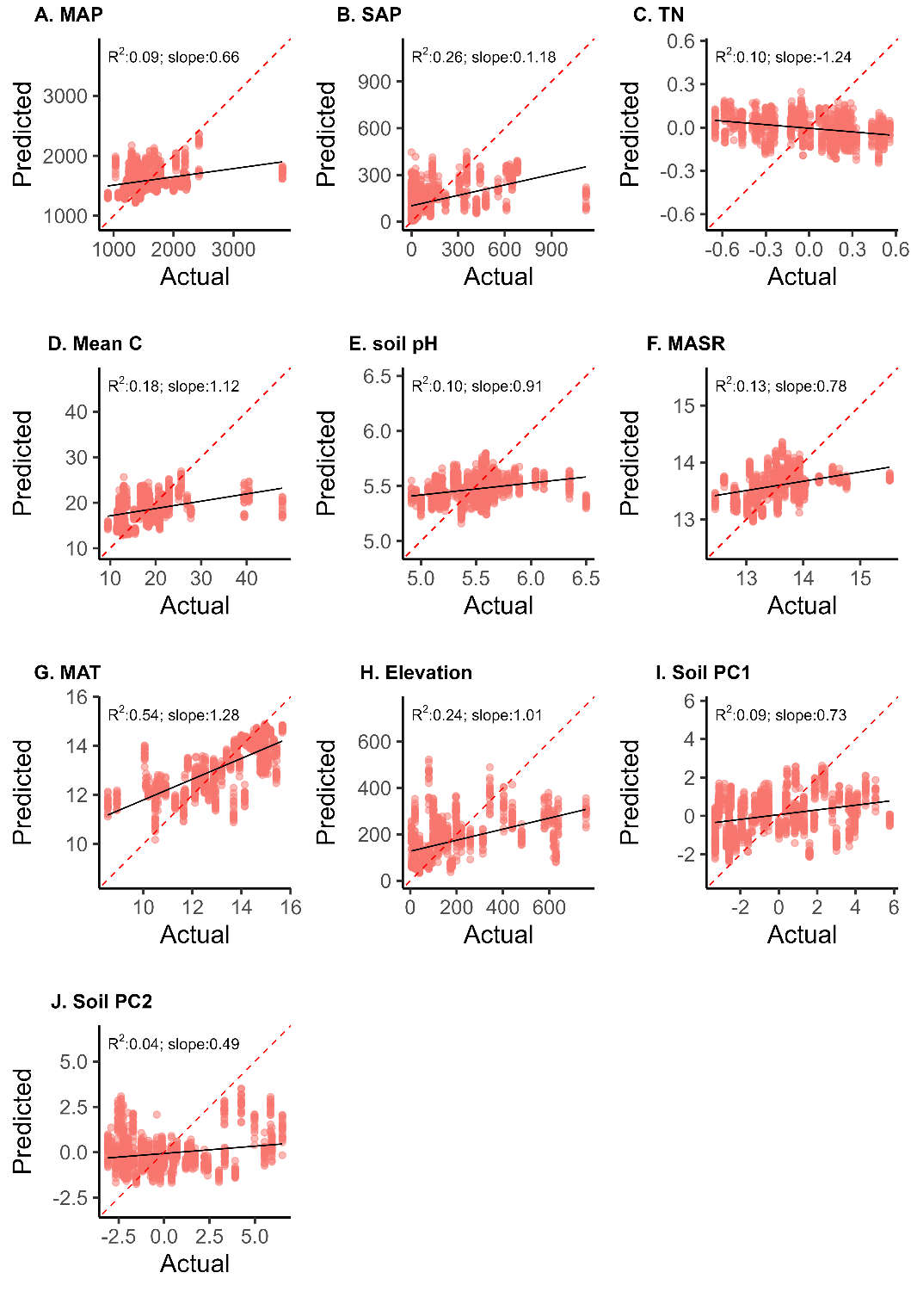


Figure S7. Accuracy of the random forest models in predicting (a-h) physicochemical variables or (i-j) PCA scores of the undisturbed sites (native sites). Dashed red lines indicate where points should fall for an exact prediction, while solid black lines represent the linear regression for each model’s predicted versus actual values. R2 and slope values are indicated in the upper left of each linear regression plot. Each plot contains predicted scores from 100 iterations of random forest models, using different randomly selected subsets of data each time. Abbreviations are detailed in the supplementary material.

**References**

Collier, K.J. (2008) Temporal patterns in the stability, persistence and condition of stream macroinvertebrate communities: relationships with catchment land-use and regional climate. Freshwater Biology 53: 603-616.

Leathwick, J.R., West, D., Gerbaeux, P., Kelly, D., Robertson, H., Brown, D. et al. (2010) Freshwater ecosystems of New Zealand (FEN) geodatabase, version 1user guide, Department of Conservation, Wellington, New Zealand.

MfE (2004) New Zealand landcover database II, User guide. Ministry for the Environment, Wellington, New Zealand.

Newsome, P., Wilde, R., and Willoughby, E. (2000) Land Resource Information System Spatial Data Layers: Landcare Research NZ Ltd., Palmerston North, New Zealand.

Wratt, D.S., Tait, A., Griffiths, G., Espie, P., Jessen, M., Keys, J. et al. (2006) Climate for crops: integrating climate data with information about soils and crop requirements to reduce risks in agricultural decision-making. Meteorological Applications 13: 305-315.
